# Improvement of the Catalytic Ability of a Thermostable and Acidophilic β-Mannanase Using a Consensus Sequence Design Strategy

**DOI:** 10.3389/fmicb.2021.722347

**Published:** 2021-09-01

**Authors:** Qingping Liang, Yuming Zhan, Mingxue Yuan, Linyuan Cao, Changliang Zhu, Haijin Mou, Zhemin Liu

**Affiliations:** ^1^College of Food Science and Engineering, Ocean University of China, Qingdao, China; ^2^Shandong Provincial Key Laboratory of Quality Safety Monitoring and Risk Assessment for Animal Products, Jinan, China

**Keywords:** β-mannanase, consensus sequence design, catalytic ability, thermostability, rational design

## Abstract

In order to improve the catalytic efficiency of a thermostable and acidophilic β-mannanase (ManAK; derived from marine *Aspergillus kawachii* IFO 4308), three mutants were designed by amino acid sequence consensus analysis with a second β-mannanase (ManCbs), which also belongs to the glycoside hydrolase family 5 (GH5) and has excellent catalytic efficiency. Three mutants were constructed and their biochemical characteristics were measured after heterologous expression in *Pichia pastoris*. The results revealed that the k_cat_/K_m_ values of the three recombinant mannanases ManAK^C292V^, ManAK^L293V^, and ManAK^L294H^ were enhanced by 303.0, 280.4, and 210.1%, respectively. Furthermore, ManAK^L293V^ showed greater thermostability than ManAK, retaining 36.5% of the initial enzyme activity after incubation at 80°C for 5min. This study therefore provides a rational design strategy based on consensus sequence analysis to develop industrially valuable β-mannanase for future applications in marine aquafeed.

## Introduction

β-Mannanases are endo-hydrolases, which can hydrolyze mannan to small molecular degradation products (Chauhan et al., 2012; Finn et al., 2014; Srivastava and Kapoor, 2017). They have been used as food stabilizers, bleaching agents in papermaking, and washing and cleaning agents (Kaira et al., 2016). They are also added into fish feedstuffs (such as soybean meal) to improve the nutritional value (Cai et al., 2011), and can significantly reduce the viscosity of coffee extract to reduce the energy consumption of large-scale coffee production (Chauhan et al., 2014a). Furthermore, manno-oligosaccharides (MOS), which are regarded as potential novel prebiotics, and are added into aquafeed, can be prepared by hydrolysis with β-mannanases (Jana et al., 2020). Therefore, improved enzymatic properties of β-mannanases, such as thermal stability, acid tolerance, and high catalytic ability would be desirable.

In our previous work, a β-mannanase (ManAK) derived from marine *Aspergillus kawachii* IFO 4308 was heterologously expressed in *Pichia pastoris* (Liu et al., 2020b). ManAK is a thermostable and acidophilic enzyme, with an optimal catalytic temperature of 80°C and pH optimum of 2.0. This characteristic makes ManAK a valuable enzyme in MOS preparation from plant gums [e.g., konjac gum, locust bean gum (LBG), and guar gum], because both high temperature and acidic conditions could decrease the stickiness of the substances and accelerate enzymatic hydrolysis (Zheng et al., 2018; La Rosa et al., 2019). However, the low catalytic efficiency of ManAK has restricted the economic production of MOS. Therefore, improvement of the catalytic efficiency of ManAK without impairing its innate thermostability and acid tolerance is meaningful.

Consensus sequence design (CSD) strategy offers a promising solution for designing enzymes of evaluated catalytic efficiency, while retaining other original properties (Sternke et al., 2019). Since the amino acid sequence of the catalytic region is generally recognized as one of the most important factors affecting the catalytic efficiency of enzymes, amino acids in the catalytic region are normally selected as the mutation targets to improve the catalytic efficiency of enzymes. Ratananikom et al. (2013), for example, reported that two β-glucosidases (Dalcochinase and Abg) had different levels of catalytic efficiency toward glycone substrates, and believed that it might be caused by the difference in amino acid residues around the glycobase-binding region. Based on this, three mutants of dalcochinase were constructed (F196H, S251V, and M369E), and the catalytic efficiency was shown to have been significantly improved. However, this CSD strategy has rarely been used to improve the catalytic efficiency of β-mannanases.

According to the classification of the carbohydrate activity database,^1^ ManAK is in the GH5 family (Larsson et al., 2006; van Zyl et al., 2010; Liu et al., 2020b). β-mannanases of this family share a similar (β/α)_8_-folded barrel structure and catalyze the reaction by complying with the double substitution reaction mechanism (Hilge et al., 1998; Zhang et al., 2008). ManCbs, derived from the fibrolytic bacterium *Cellulosimicrobium* sp. strain HY-13, is a neutral-type mannanase in the same family, but has much higher catalytic activity than ManAK. The specific activity of ManCbs can be as high as 14,711IU mg^−1^ (Kim et al., 2011), which is greater than that of ManAK. Considering that ManAK and ManCbs share high sequence similarity (86.4%) in the catalytic region, it is theoretically feasible to enhance the catalytic efficiency of ManAK according to CSD strategy.

In this study, the CSD strategy was adopted to rationally design potential enzyme mutants with elevated catalytic efficiency. Three recombinant ManAK mutants were heterologously expressed by *P. pastoris*. The recombinant enzymes were purified, and their enzymatic properties were fully assayed. Changes in enzymatic properties were illustrated in terms of the structure-function relationship by analyzing the alterations in non-covalent bond interactions between ManAK and its mutants.

## Materials and Methods

### Materials

Thermostable and acidophilic β-mannanase was purified from *A. kawachii* IFO 4308 (BioProject accession: PRJDA66971), and the coding sequence was derived from the gene sequence published by NCBI with the GenBank accession No.: GAA89125.1 as previously reported (Liu et al., 2020b). The *Escherichia coli* DH5α and the *P. pastoris* X33 (Invitrogen, United States) were purchased from Sigma-Aldrich (St. Louis, MO, United States). These strains were used to prepare expression plasmids and as expression hosts, respectively. All chemicals and reagents used were purchased from Sigma-Aldrich (St. Louis, MO, United States) and of analytical grade.

### Alignment Analysis of Gene Sequences of ManAK and ManCbs

Thermostable and acidophilic β-mannanase and ManCbs belong to the GH5 family, and the two gene sequences are highly conserved and homologous. The tertiary structure of ManAK was automatically modeled by the Phyre2 program^2^ based on the reported crystal structure of ManBK (PDB code: 3WH9; 95% identity), and the tertiary structures of ManCbs were modeled by the same way. Sequence alignment and structure analysis were performed by Discovery Studio 2.5 software (Accelrys, San Diego, CA, United States). Figure rendering was performed by PyMOL software (version 1.7.2; Delano Scientific, United States).

### Construction of Recombinant Expression Plasmids of ManAK

Three mutants (ManAK^C292V^, ManAK^L293V^, and ManAK^L294H^) were created based on the amino acid sequence consensus analysis between ManAK and ManCbs. Vector NTI 13.5 was used to design and analyze the PCR mutation primers according to the rational design sequence of ManAK. To insert the mutated gene into the expression vector pPICZα A, all primers had homology arm sequences at both ends of the vector insertion site (Table 1). The T_m_ values of all primers were approximately 60°C, and GC% was kept below 60%. Both primers were synthesized by the Ruibo Xingke Company, Beijing.

**Table 1:** Primers used for site-directed mutagenesis.

Mutants	Primers
ManAK^C292V^	Reverse: 5'-TGGTAAGCCAGTTTTGTTGGAAGAATACGGTGTTACTTCTAACCAT-3' Forward: 5'-CTTCCAACAAAACTGGCTTACCAGCAGCCTTACAAGC-3'
ManAK^L294H^	Reverse: 5'-TAAGCCATGTGTTTTGGAAGAATACGGTGTTACTTCTAACCATTG-3' Forward: 5'-ATTCTTCCAAAACACATGGCTTACCAGCAGCCTTACAAG-3'
ManAK^L294H^	Reverse: 5'-GCCATGTTTGGATGAAGAATACGGTGTTACTTCTAACCATTGTTCT-3' Forward: 5'-CGTATTCTTCATCCAAACATGGCTTACCAGCAGCCTTAC-3'

pPICZα A-AK was constructed by ligating the original ManAK gene (GenBank accession No.: GAA89125.1) into the pPICZα A expression plasmid, and the integrated vector was transformed into *E. coli* DH5α, which was considered as the template for the preparation of the other three recombinant expression plasmids. Synthetic primers were used to amplify the three mutant enzyme genes by PCR reaction according to the Mut Express® II kit instructions. The three recombinant expression plasmids were constructed by homologous recombination of the genes prepared with the pPICZα A expression plasmid; named pPICZα A-ManAK^C292V^, pPICZα A-ManAK^L293V^, and pPICZα A-ManAK^L294H^.

### Heterologous Expression of Recombinant Mutants in *P. pastoris* and Purification

The recombinant expression plasmids pPICzα A-ManAK^C292V^, pPICzα A-ManAK^L293V^, and pPICzα A-ManAK^L294H^ were linearized and digested with Sal I enzyme, and transformed into *P. pastoris* X33 by electroporation. The transformed colonies were grown on YPD solid plates containing 100μg/ml Zeocin (Thermo Scientific) and incubated at 30°C for 5days. Colonies were then transferred to Buffered Minimal Glycerol-complex Medium, and 1.0% (v/v) methanol was added to induce protein expression. The cultures were incubated in a shaker at 30°C for 3days to allow secretion of the expression product. Supernatant was collected and concentrated by ultrafiltration with a 50kDa membrane. The sample was then loaded onto a Ni-Sepharose 6FF column (GE Healthcare, United States), and the sample eluted according to the method described by Zhang et al. (2019). The sample was dialyzed against 50mM potassium phosphate buffer (pH 5.5) and analyzed by SDS-PAGE gel chromatography. The concentration of the purified enzyme solution was determined by the Bradford method (Bradford, 1976).

### Biochemical Characterization of Recombinant ManAK^C292V^ and ManAK^L293V^ and ManAK^L294H^

To assess the effects of the site directed mutagenesis, the biochemical characteristics of the recombinant enzymes were measured. The enzymatic activity of mannanases was determined using the 3,5-dinitrosalicylic acid (DNS) method: the expressed enzyme solution was diluted with 50mM sodium acetate buffer, and reacted with an equal volume of 0.5% (w/v) D-mannan (Sigma, St. Louis, MO, United States) at 37°C for 30min. The D-mannan used here was LBG (Sigma), which was dissolved in boiling sodium acetate buffer, and then diluted to 100ml to prepare the substrate. Subsequently, DNS reagent was added and the reaction incubated in a boiling water bath for 5min, and then the optical density measured at 540nm (Miller, 1959). One unit (U) of mannanase activity is defined as the amount of enzyme needed to generate 1μmol of reducing sugar/minute at pH 5.5 and 37°C from a solution of 3mg/ml mannan.

The optimal temperature for activity of the mannanases was determined by comparing the relative enzyme activity of the sample at a range of temperatures between 35 and 85°C (in 50mM sodium acetate buffer, pH 5.5). The mixture samples of enzymes and substrate were reacted at various temperatures for 5min to access the recombinant mannanase activity. In addition, the thermal stability of the enzymes was determined after preincubation of enzymes without substrate in 50mM sodium acetate buffer (pH 5.5) at different temperatures (60, 70, and 80°C) for different times (5, 10, 15, 20, and 25min). After this thermal treatment, the enzymes were mixed with substrate and reacted under standard conditions (pH 5.5, 37°C, and 30min) to obtain the residual rate of enzyme activity after heat treatment.

Dilute mannanase enzyme solution was tested with the different pH buffers (i) Glycine-HCl (pH 1.0–4.0), (ii) sodium acetate buffer (pH 4.0–6.0), and (iii) Tris-HCl buffer (pH 6.0–8.0). Using the 0.5% (w/v) LBG under different pH conditions (1.0–8.0) as substrates, the optimal pH of recombinant mannanases was determined under standard conditions (37°C, 30min). After the sample was treated with a buffer of the same pH value range (1.0–8.0) at 37°C for 2h, the residual rate of enzyme activity was measured according to the same method to obtain the pH stability.

To obtain the kinetic characteristics of the mannanase samples, the enzyme activity was measured under standard conditions using 1–10mg/ml LBG as a substrate. The data were analyzed by Origin 8.5 software and the dynamic parameters K_m_ and k_cat_ were obtained. Dynamic curves were drawn according to the nonlinear regression method.

The three-dimensional structures of ManAK and recombinant mutants were modeled by the Phyre2 website (see Footnote 2; Kelley et al., 2016). Using Auto Dock Tools 1.5.6, the three recombinant samples were docked by molecular simulation and compared. The differences between the structure of the native ManAK and the modified versions around the mutated sites were then obtained. The software used in the molecular visualization and graphics drawing process was PyMOL (v1.7.2; Delano Scientific, United States), and Discovery Studio 2.5 software was used to make a simulated two-dimensional diagram of the interaction force between molecules.

### Analysis of Hydrolysis Products

In order to understand and compare the degradation efficiency and mode of action of ManAK and its mutant forms, the hydrolysis products of the mannobiose (M2), mannotriose (M3), mannotetraose (M4), and mannopentaose (M5) standard of MOS were analyzed using the ManAK^L293V^ mutant. The reaction was carried out at 37°C for 10min for all MOS, and the reactants were 5U purified enzyme and 20mM MOS standard solution. The reaction was terminated by boiling the sample in water for 10min, then the macromolecules were removed using a 3-kDa Millipore membrane (Millipore, United States) for ultrafiltration. After centrifugation at 13,000*g* for 10min, supernatants were collected and analyzed according to the procedure as previously described (Katrolia et al., 2013), using TLC plate (Silica gel 60F254, Merck, Darmstadt, Germany) to analyze the samples.

The hydrolysis products of recombinant ManAK and the mutant ManAK^L293V^ were further analyzed using high-performance liquid chromatography [HPLC; Agilent 1260 Infinity system TSKgel G-oligo-PW column (7.5mm×300mm), Tosoh Corporation Co. Japan]. Purified ManAK and ManAK^L293V^ were diluted with 50mM sodium acetate buffer (pH 5.5) to give a final enzyme activity of 5U, and reacted with 20mM MOS standard solution (M4 and M5) at 37°C for 20min. The products were detected with ultra-pure water as the mobile phase at a flow rate of 0.6ml/min. All samples were injected after filtering through a 0.22μm filter membrane, and the hydrolysis products were analyzed sequentially.

The release of reducing sugar was determined as follows: 5U purified enzyme and 0.5% (w/v) D-mannan (Sigma, United States) in 50mM sodium acetate buffer (pH 5.5) were incubated at 37°C for varying lengths of time, and the concentration of released reducing sugars measured by the DNS method as above.

To explore the application of this β-mannanase, the recombinant ManAK and the ManAK^L293V^ mutant were assessed for their hydrolytic performance, using LBG as a substrate. LBG was dissolved in 50mM sodium acetate buffer (pH 5.5) to give a final concentration of 1.0%, and purified ManAK and ManAK^L293V^ were diluted with 50mM sodium acetate buffer (pH 5.5) to 5 enzyme activity units. The reaction was carried out at 37°C for 12h. Molecular weight distribution of the hydrolysis products was detected by the HPLC [Agilent 1260 Infinity system TSKgel G4000PWXL column (7.8mm×300mm), Tosoh Corporation Co. Japan]. The mobile phase of the detection system was 0.2M NaNO_3_ and 0.01M NaH_2_PO_4_, and the flow rate was 0.2ml/min.

### Statistical Analysis

All experimental results were repeated three times, and they were statistically analyzed using ANOVA (*t*-test) with IBM SPSS Statistics 26 software. Differences with a threshold of *p*<0.05 were considered statistically significantly.

## Results

### Sequence Alignment Analysis of ManAK and ManCbs Based on CSD Strategy

Analysis of the conserved domains of the gene sequences of ManAK and ManCbs showed that they both belonged to the GH5 family, and that the proportion of amino acid sequence homology in the catalytic region was as high as 86.4%. Two key amino acids (Glu188 and Glu296) in the catalytic sites of ManAK exist as proton donors and nucleophiles, respectively, which play an important role in hydrolysis (Zhang et al., 2019). When ManAK binds to the substrates, a catalytic cleft is formed between the two catalytic domains (Glu188 and Glu296), and the amino acid residues around them assist with hydrolysis (Hilge et al., 1998).

**Figure 1:**
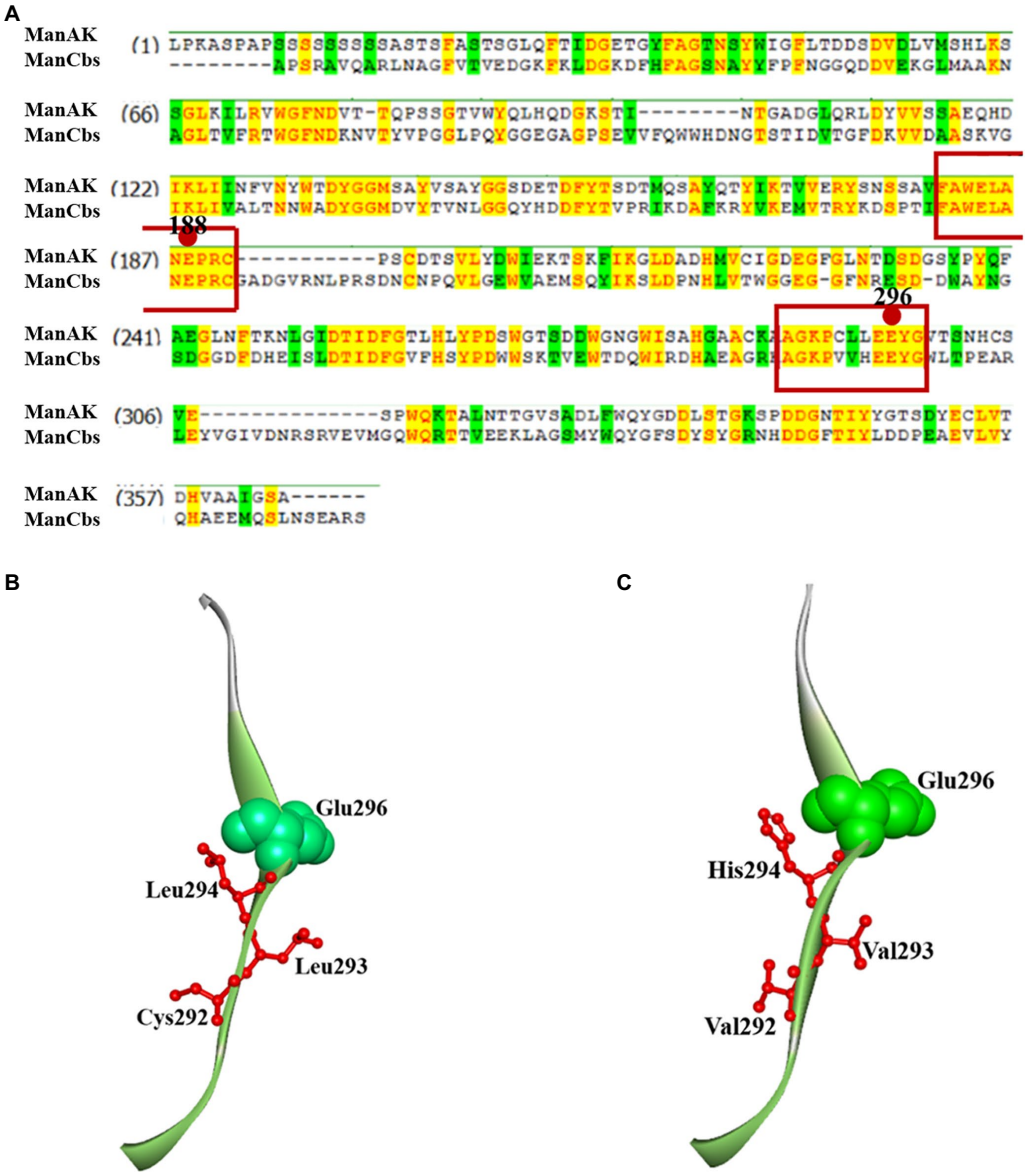
Amino acid consensus sequence analysis and secondary structure around E296 of thermostable and acidophilic β-mannanase (ManAK) and ManCbs. **(A)** Amino acid consensus sequence analysis of ManAK and ManCbs. Strictly conserved residues are shaded yellow. The active site residues in catalytic regions are boxed and circles indicate the catalytic sites in catalytic region. **(B)** Secondary structure around E296 of ManAK. **(C)** Secondary structure around E296 of ManCbs. The amino acids in position 292, 293, and 294 are indicated in red, and E296 is indicated in green balls.

The sequence comparison also showed that the amino acid sequence of the catalytic region around Glu188 was highly consistent (Figure 1). However, the amino acids around Glu296 in ManAK (Cys292, Cys293, and Leu294) were different from those in ManCbs (Val292, Val293, and His294). Combined with the structural analysis of ManAK and ManCbs in the catalytic region of Glu296, it was shown that the three different amino acids in positions 292, 293, and 294 were in the same β-sheet structure, and were adjacent to Glu298 (Figures 1B,C). The amino acid sequence of the three sites is likely to have a significant impact on the catalytic efficiency of the enzyme. Thus, through consensus sequence analysis, and structure analysis on the different amino acids in catalytic region, three mutants (ManAK^C292V^, ManAK^L293V^, and ManAK^L294H^) were constructed to assess the underlying influences of the three amino acids on the catalytic efficiency of ManAK.

### Kinetic Parameters and Catalytic Efficiency of Recombinant Mannanases

The recombinant plasmids pPICZα A-ManAK^C292V^, pPICZα A-ManAK^L293V^, pPICZα A-ManAK^L294H^ were transformed into the expression host *P. pastoris* X33. SDS-PAGE showed that the detected molecular weights of recombinant enzymes were approximately 50kDa.

Locust bean gum was used as a substrate to determine the kinetic parameters of ManAK and its mutants. According to the Michaelis-Menten equation, the results demonstrated that the V_max_ and K_m_ values of ManAK were 329.3U/mg·min and 1.09mg/ml, and the catalytic efficiency k_cat_/K_m_ value was 199.3ml/s·mg (Table 2). Although, the V_max_ of the three recombinant mannanases was not obviously improved, the K_m_ values were changed from 1.09mg/ml (ManAK) to 0.10mg/ml (ManAK^C292V^), 0.14mg/ml (ManAK^L293V^), and 0.07mg/ml (ManAK^L294H^), which indicated that the substrate affinity of the three mutants was improved.

**Table 2:** Kinetic parameters of ManAK, ManAK^C292V^, ManAK^L293V^, and ManAK^L294H^.

	V_max_ (U/mg·min)	K_m_ (mg/ml)	K_cat_ (S^−1^)	K_cat_/K_m_ (ml/s·mg)
ManAK^C292V^	126.80±8.29	0.10±0.09	83.67	803.22
ManAK^L293V^	154.22±12.32	0.14±0.12	101.72	758.07
ManAK^L294H^	64.29±20.52	0.07±0.18	42.41	618.01
ManAK	329.30±25.50	1.09±0.28	217.21	199.30

The mutated amino acids, Val292, Val293, and His294, were shown to greatly affect the catalytic efficiency of ManAK. The results revealed that the k_cat_/K_m_ values of the three recombinant mannanases ManAK^C292V^, ManAK^L293V^, and ManAK^L294H^ were enhanced by 303.0, 280.4, and 210.1%, respectively (Table 2). This proved that the CSD strategy was effective in improving the catalytic efficiency of mannanase. Further study is needed to show whether other properties of the enzyme could be similarly improved using this modification strategy.

### Other Biochemical Characteristics of ManAK and Mutants

**Figure 2:**
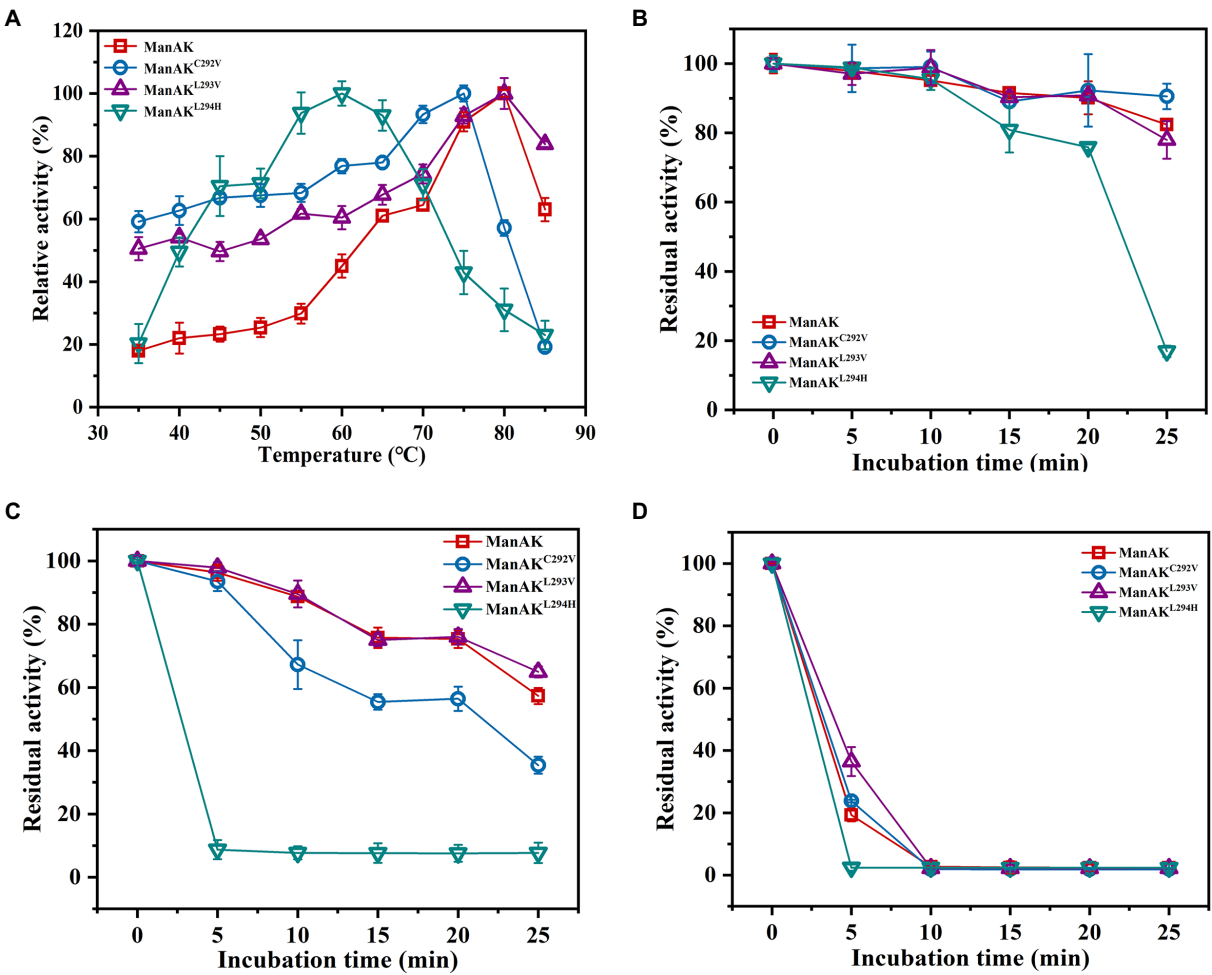
Effects of temperature on activity and stability of recombinant β-mannanases. **(A)** The optimum temperature was determined in 50mM sodium acetate buffer (pH 5.5) with a range of 35–85°C for 5min; the thermal stability was evaluated under standard conditions (pH 5.5, 37°C, and 30min) after the enzymes without substrate were treated at 60 **(B)**, 70 **(C)**, and 80°C **(D)** for 5–25min.

Thermostable and acidophilic β-mannanase showed excellent temperature characteristics with an optimal temperature for activity of 80°C (Figure 2A); and even retained approximately 19.3% of enzyme activity after incubation at 80°C for 5min (Figure 2D). Three mutants of ManAK were constructed and their biochemical characteristics were accessed. While the catalytic efficiency was improved, the thermal stability of ManAK^L293V^ was further enhanced. Compared with the original enzyme ManAK, the optimum temperature of ManAK^L293V^ was unchanged, remaining at 80°C, but that of ManAK^C292V^ and ManAK^L294H^ dropped to 75 and 60°C, respectively (Figure 2A). Interestingly, after heating at 80°C for 5min, ManAK^L293V^ retained 36.5% of the initial enzymatic activity, while ManAK retained only 19.3% (Figure 2D).

**Figure 3:**
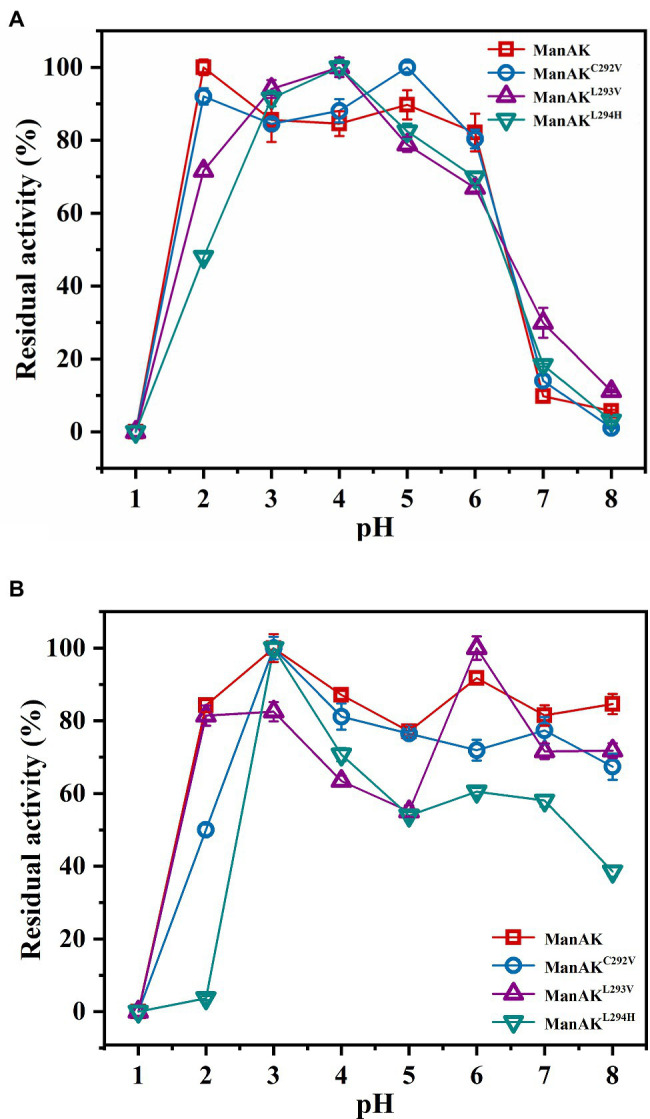
Effects of pH on activity and stability of recombinant β-mannanases. **(A)** The pH optimum was determined in glycine–HCl buffer (pH 1.0–4.0), sodium acetate buffer (pH 4.0–6.0), and Tris-HCl buffer (pH 6.0–8.0), respectively. **(B)** The pH stability was determined after the enzymes were incubated at 37°C for 2h in different buffers as described above.

The ManAK is an extremely acidophilic mannanase with an optimal pH of 2.0 (Figure 3A), and after treatment in a buffer solution at pH 2.0, 84.3% relative enzymatic activity of ManAK was retained. Similarly, the pH profiles of the three mutants revealed that they retained strong activity under acidic conditions, and the optimal pH was acidic (Figure 3A). However, ManAK^L294H^ only retained 3.68% activity after treatment in a buffer solution at pH 2.0, possibly because H294 is a basic amino acid (Figure 3B).

### Analysis of Hydrolysis Products

**Figure 4:**
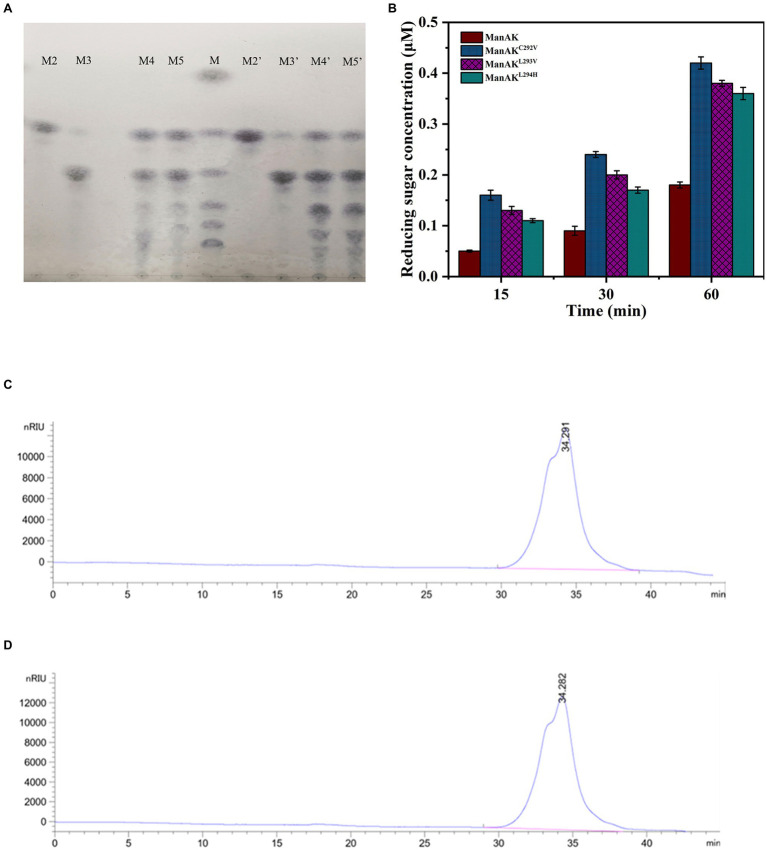
Analysis of hydrolysis products. **(A)** The hydrolysis of substrates by ManAK and ManAK^L293V^, and thin-layer chromatography (TLC) analysis of hydrolysis products from manno-oligosaccharides (MOS) standards (M2–M5). The reaction was carried out at 37°C for 10min, and the reactants were 5U purified enzyme and 20mM MOS standard solution. Lanes: (M) the MOS standards consist of mannose, mannobiose, manninotriose, mannotetrose, mannopentose, and mannohexose (from top to bottom; M2–M5) the hydrolysis products of mannobiose, manninotriose, mannotetrose, and mannopentose, respectively, (M2’–M5’) the same indication as above, analysis of hydrolysis products of ManAK^L293V^. **(B)** The release of reducing sugar concentration of recombinant mannanases incubated with D-mannan in 50mM sodium acetate buffer (pH 5.5) at 37°C. **(C)** High-performance liquid chromatography (HPLC) analysis of the molecular weight distribution of hydrolysis products from locust bean gum (LBG) by ManAK (reaction carried out at 37°C for 12h). **(D)** HPLC analysis of the molecular weight distribution of hydrolysis products from LBG by ManAK^L293V^ (reaction carried out at 37°C for 12h).

In order to verify whether the hydrolytic performance of the recombinant mannanase was affected, the hydrolysis products from the mutant ManAK^L293V^ and ManAK were compared. The results showed that there was no significant difference between the hydrolysis products (Figures 4A,C,D), indicating that the site-mutation of L293V had no negative influence on hydrolysis.

The enzymes were unable to cleave M2 and showed very limited cleavage of M3, which indicated that ManAK was an endo-mannanase (Figure 4A). The enzymes were able to efficiently hydrolyze M4 and M5, proving that the enzyme was useful for the preparation of prebiotic oligosaccharides. In addition, M5 sugars were detected during the hydrolysis of M4 (Figure 4A), showing that ManAK and ManAK^L293V^ shared similar transglycosylation activity. Therefore, the modification strategy used in this work did not affect the hydrolysis mode.

According to the results of the TLC analysis, M4 and M5 were selected as substrates to further analyze the hydrolysis products by the HPLC. The results were consistent with the results of TLC analysis. As show in Table 3, both ManAK and ManAK^L293V^ can effectively degrade M4 and M5 to generate small molecule oligosaccharides, and M5 can be detected during the degradation of M4, and M6 can be detected during the degradation of M5. Furthermore, when degrading M4 and M5, the main products generated by ManAK were M3, with a composition of 42.9 and 36.4%, respectively.

**Table 3:** High-performance liquid chromatography analysis of the hydrolysis products of mannan polymers by ManAK and ManAK^L293V^.

Substrate	Composition (%) of products formed by hydrolysis reaction						M1	M2	M3	M4	M5	M6
**ManAK**						
M4	6.3	20.9	42.9	23.6	4.9	1.2
M5	16.4	21.1	36.4	17.5	8.1	0.4
**ManAK^L293V^**						
M4	7.2	22.4	43.5	20.1	6.2	0.6
M5	15.4	25.6	38.2	13.1	6.9	0.8

To better understand the improvement in catalytic efficiency of the three recombinant mannanases, the reducing sugars released were measured under the same conditions. The results demonstrated that the yields of reducing sugars of the different mutants were all higher than that of ManAK, which corresponded with the results of the kinetic analysis (Figure 4B).

Locust bean gum was used as a substrate to illustrate the application potential of ManAK and the mutant ManAK^L293V^. The results showed that they can effectively degrade LBG (Figures 4C,D), and the products hydrolyzed were oligosaccharides with a molecular weight below 3,000Da, which indicated that ManAK had application potential in the preparation of MOS.

### Analysis of Structures and Interaction Forces in Mutated Sites of ManAK and Mutants

**Figure 5:**
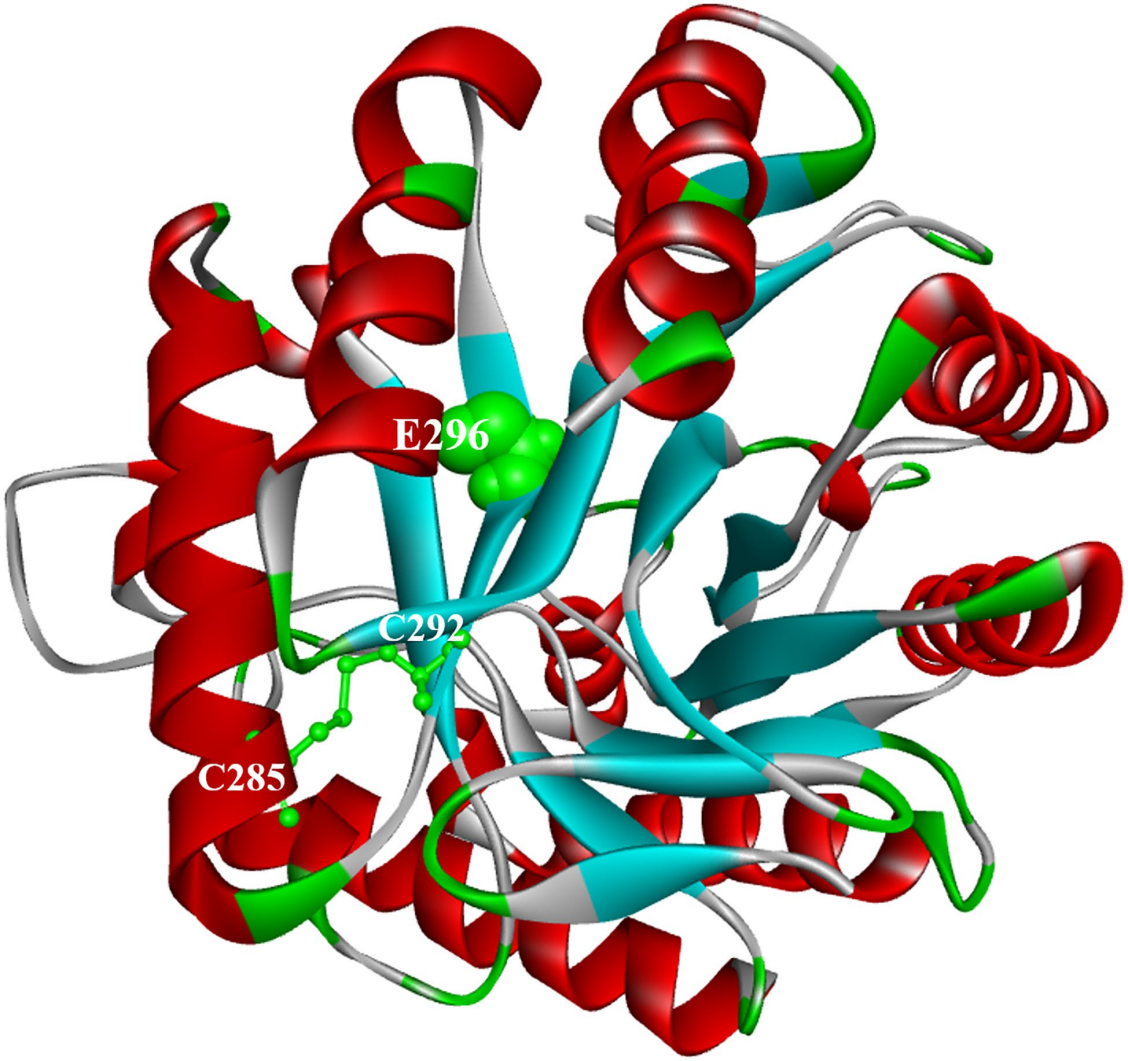
Docking analysis and overall structure of ManAK. The catalytic region around E296 is illustrated in ball forms and colored in green. The disulfide bond formed between C292 and C285 is shown as stick models and colored in green.

**Figure 6:**
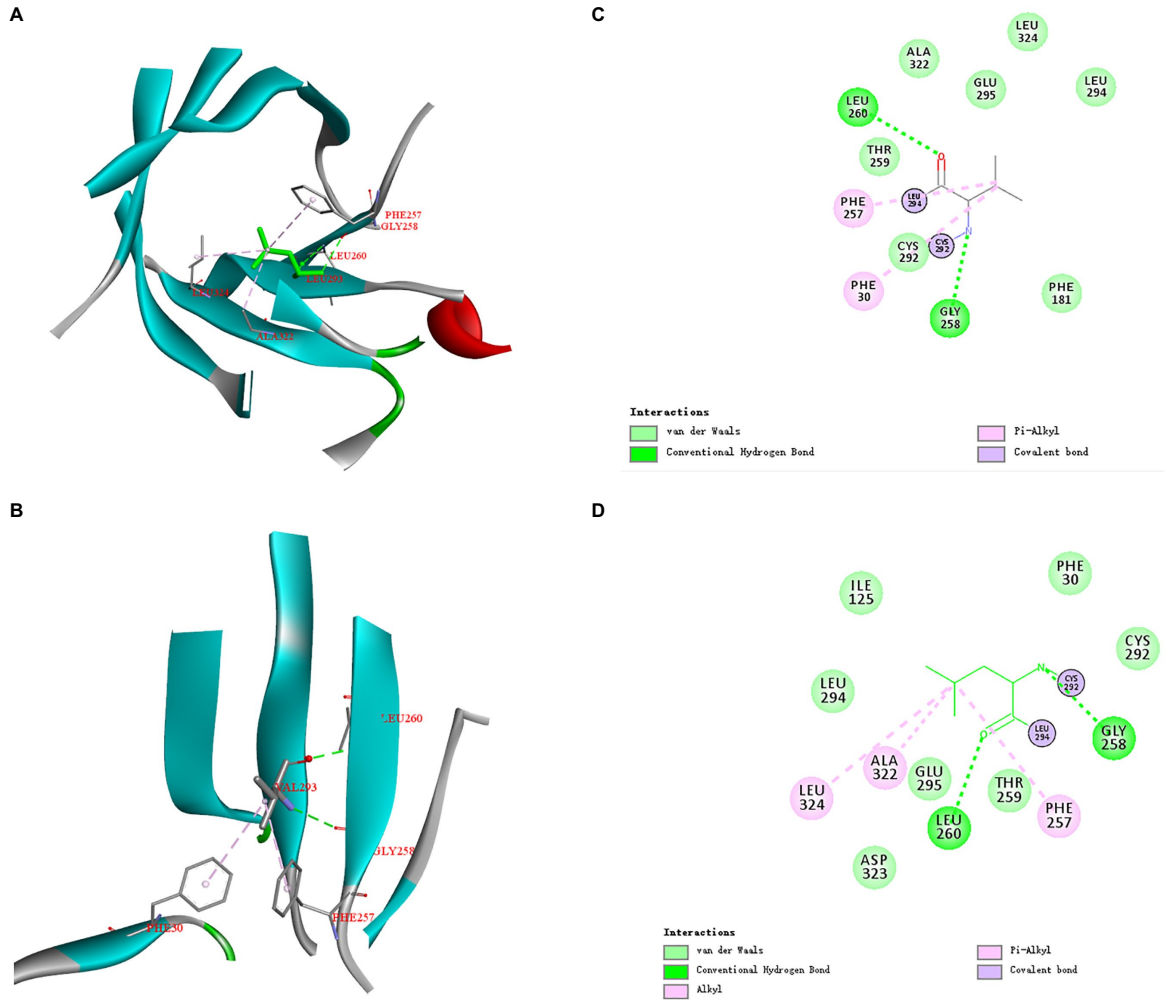
Overall structure and modeled complex structures of ManAK **(A)** and ManAK^L293V^ **(B)** in position 293. The amino acid residues around position 293 are indicated in red. The interactions between them are represented by a dashed line. Interaction analysis of amino acid site ManAK **(C)** and ManAK^L293V^ **(D)**. The green dashed line indicates Conventional Hydrogen Bond interaction; the pink dashed line indicates Alkyl and Pi-Alkyl interaction. Amino acid residues that form van der Waals interactions with amino acid in position 293 are indicated in light green balls and Covalent bond are indicated in purple.

**Figure 7:**
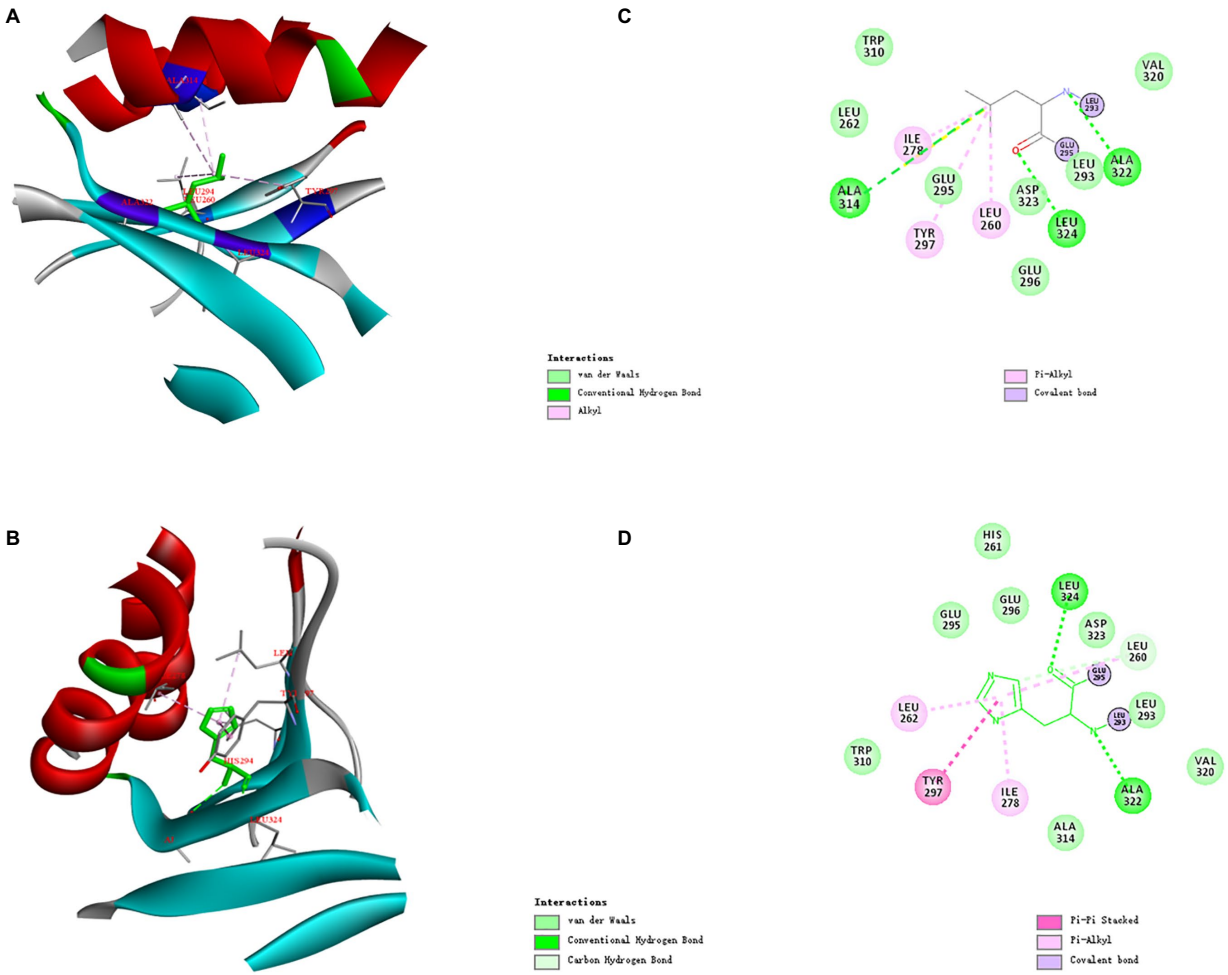
Overall structure and modeled complex structures of ManAK **(A)** and ManAK^L294H^ **(B)** in position 294. The amino acid residues around position 294 are indicated in red. The interactions between them are represented by a dashed line. Interaction analysis of amino acid site ManAK **(C)** and ManAK^L294H^ **(D)**. The green dashed line indicates Conventional Hydrogen Bond interaction; the pink dashed line indicates Alkyl and Pi-Alkyl interaction; the deep pink dashed line indicates Pi-Pi stacked interaction. Amino acid residues that form van der Waals interactions with amino acid in position 294 are indicated in light green balls and Covalent bond are indicated in purple.

The three-dimensional structures of ManAK and recombinant mutants were modeled. The results showed that the structures and interaction forces of the three mutants at the mutated sites were changed compared with those of ManAK (Figures 5–7). The modeled three-dimensional structure of ManAK indicated that a disulfide bond was formed between C292 and C285 (Figure 5). The ManAK^C292V^ mutation destroyed the disulfide bond seen in the original enzyme. As shown in Figure 6, ManAK and ManAK^L293V^ both had a parallel folded β-sheet, while ManAK^L293V^ had a newly force (Alkyl) compared with the original interaction forces (van der Waals, Pi-Alkyl, Conventional Hydrogen Bond, and Covalent bond) seen in ManAK. The modeled structures of ManAK^L294H^ and ManAK in position 294 showed that they all involved in the secondary structure of α-helix and β-sheet (Figures 7A,B). However, the results of analysis of interaction forces at the 294 position demonstrated that the mutant ManAK^L294H^ had a newly introduced Pi-Pi stacked interaction and one less conventional hydrogen bond than ManAK (Figures 7C,D).

## Discussion

Since β-mannanase has excellent potential in the production of industrial feedstuff, its catalytic efficiency has garnered attention. In this study, CSD strategy, a method previously used to improve the catalytic efficiency of β-glucosidases (Ratananikom et al., 2013), was applied to ManAK. The K_m_ values of the three mutants were reduced compared with ManAK, which demonstrated that mutations (C292V, L293V, and L294H) could improve the substrate affinity of ManAK. The K_m_ values of the mutants were also lower than those of most reported mannanases, such as those from *Bacillus* sp. HJ14 (2.20mg/ml; Zhang et al., 2016), *Phialophora* sp. P13 (2.50mg/ml; Zhao et al., 2010), *Bacillus* sp. JAMB-602 (3.10mg/ml; Takeda et al., 2004), *Bacillus halodurans* PPKS-2 (3.85mg/ml; Vijayalaxmi et al., 2013), *Phialophora pinophilum* C1 (5.60mg/ml; Cai et al., 2011), *Bacillus subtilis* BE-91 (7.14mg/ml; Cheng et al., 2016), *Bacillus nealsonii* PN-11 (7.22mg/ml; Chauhan et al., 2014b), *B. subtilis* WY34 (7.60mg/ml; Jiang et al., 2006), *B. subtilis* MAFIC-S11 (8.00mg/ml; Lv et al., 2013), *Bacillus* sp. MSJ-5 (11.67mg/ml; Zhang et al., 2009), and *B. subtilis* YH12 (30.00mg/ml; Liu et al., 2015).

The k_cat_/K_m_ of the three mutants was significantly improved compared to ManAK. This was superior to the previously reported catalytic efficiency of mannanases, such as those from *Penicillium canescens* (Sinitsyna et al., 2008) and *Rhizomucor miehei* (Katrolia et al., 2012). The substrate affinity of the three mutants was greatly enhanced due to the significant decrease in K_m_ values, which might increase the k_cat_/K_m_ values. However, the improvement in catalytic efficiency may be limited. With the constant increase in substrate concentration, the advantage of high catalytic efficiency may be reduced. In fact, within a certain range of substrate concentrations, these three recombinant mannanases can show superior catalytic efficiency. Moreover, interestingly, even though these three mutants all displayed elevated catalytic efficiencies, changes in their thermostability were either improved (ManAK^L293V^), remained the same (ManAK^C292V^), or decreased (ManAK^L294H^).

Nowadays, molecular simulation is widely used to infer the changes of non-covalent forces in the mutation site region before and after the alteration, which is helpful to explain the reasons for the changes in the thermostability and the catalytic efficiency of mutants. Molecular simulation can be used for most enzymes, including mannanases (Chauhan et al., 2015; Chauhan and Jaiswar, 2017; Liu et al., 2020a), xylanase (Cheng et al., 2015), and β-glucosidases (Dadheech et al., 2019). Therefore, in this study, the structure-function relationships of the three mutants were analyzed by docking analysis and a molecular simulation method.

ManAK^C292V^ showed elevated catalytic efficiency, which was increased by 303.0% compared with the starting enzyme ManAK, and a reduced K_m_ value. The modeled three-dimensional structure of ManAK indicated that a disulfide bond was formed between C292 and C285 (Figure 5). After the mutation, amino acid V292 destroyed the original disulfide bond, which in turn affected the original spatial structure. ManAK^C292V^ eliminated the disulfide bond to provide more flexibility at the active site, and the flexibility of the β-sheet in the catalytic region E296 was also increased, possibly increasing the affinity between enzyme and substrate. However, the thermal stability of ManAK^C292V^ was reduced (Figures 2A,C), possibly because the disulfide bond contributes to the maintenance of the stability of the enzyme.

The mutant ManAK^L293V^ showed increased catalytic activity, suggesting the importance of residue V293. The alkyl side chain of V293 was smaller than that of L293, which meant that the steric hindrance of this amino site was smaller, indicating that it provided a larger space for substrate entry and product release. Interestingly, mutant ManAK^L293V^ also showed improved thermal stability. No significant difference was detected in the spatial structure at this position between ManAK and ManAK^L293V^, with both having a parallel folded β-sheet (Figures 6A,B). However, a newly formed interaction force between alkyl groups (Figures 6C,D) might have a positive influence on the thermal stability of enzyme, which meant that V293 was thought to be the critical site for the improvement of stability.

Similar results for ManAK^L294H^ were observed in both the kinetic study and catalytic ability; it displayed a lower K_m_ value and higher catalytic efficiency, which meant the substrate-binding affinity was greatly increased. Residue Y297 of ManAK is located adjacent to the key catalytic amino site E296, and although, it might not directly interact with the substrate it generated a π-π stacking interaction with the new corresponding residue of H294 (Figure 7D). The substitution of H294 has a heteroaryl structure, which tends to form the π-π stacking interaction with the substrates, thus promoting the substrate-binding affinity and catalytic efficiency. Similar cases have been reported in previous studies in which a new stacking force is formed by the aryl of the mutant amino acid, and is conducive to substrate entry (Huang et al., 2014; Cheng et al., 2015). In contrast to the positive results, ManAK^L294H^ showed a drastically reduced optimum temperature and thermal stability (Figures 2A–D). From the complex structure model in 294 position, ManAK had a conventional hydrogen bond between L294 and A314 (Figure 7C), and involved in α-helix and β-sheet (Figure 7A). In ManAK^L294H^, the conventional hydrogen bond was destroyed and the secondary structure of α-helix was excluded (Figure 7B), indicating that the conventional hydrogen bond between L294 and A314 is indispensable for the stability of ManAK. The destruction and disappearance of this original force may have a negative impact on the stability of the protein itself.

## Conclusion

A thermostable and acidophilic β-mannanase, ManAK, derived from marine *Aspergillus kawachii* IFO 4308 would be especially suitable for MOS preparation, except for its low catalytic efficiency. To compensate for this defect, a CSD method was adopted to engineer the catalytic efficiency of ManAK. Three positive mutants (ManAK^C292V^, ManAK^L293V^, and ManAK^L294H^) showing increased k_cat_/K_m_ values were generated. Minor changes were detected in their optimal pH values and acid tolerance, while their thermostabilities varied. The mechanisms underlying the stability and catalytic efficiency were investigated, which provided an intuitive understanding of structure-function relationships. Most importantly, a superior mutant (ManAK^L293V^) with both enhanced catalytic efficiency and thermostability was acquired, which can further advance the application of ManAK in MOS preparation.

## Data Availability Statement

The original contributions presented in the study are included in the article/Supplementary Material, further inquiries can be directed to the corresponding authors.

## Author Contributions

QL contributed to the experiment planning and conduct, writing – original draft preparation, and methodology. YZ and MY also contributed to experiment conduct and revision of the writing – original draft. LC contributed to data collation and analysis, graphics drawing, and statistical analysis. CZ contributed to write and revise the manuscript. ZL contributed to software application, conceptualization, and data curation. HM contributed to writing-review, supervision, funding acquisition, and project administration. All authors contributed to the article and approved the submitted version.

## Conflict of Interest

The authors declare that the research was conducted in the absence of any commercial or financial relationships that could be construed as a potential conflict of interest.

## Publisher’s Note

All claims expressed in this article are solely those of the authors and do not necessarily represent those of their affiliated organizations, or those of the publisher, the editors and the reviewers. Any product that may be evaluated in this article, or claim that may be made by its manufacturer, is not guaranteed or endorsed by the publisher.

## Footnotes

^1^ http://www.cazy.org

^2^ http://www.sbg.bio.ic.ac.uk/phyre2/html/page.cgi?id=index
